# Combined quality of life and survival for estimation of long-term health outcome of patients with stroke

**DOI:** 10.1186/s12955-022-01959-1

**Published:** 2022-03-24

**Authors:** Nipaporn Butsing, Mathuros Tipayamongkholgul, Jung-Der Wang, Disya Ratanakorn

**Affiliations:** 1 Ramathibodi School of Nursing, Faculty of Medicine Ramathibodi Hospital, Mahidol University, Bangkok, Thailand; 2 Department of Epidemiology, Faculty of Public Health, Mahidol University, 420/1 Ratchawithi Road, Ratchathewi, Bangkok, 10400 Thailand; 3 Department of Public Health, National Cheng Kung University, College of Medicine, Tainan City, Taiwan; 4 Department of Medicine, Faculty of Medicine, Ramathibodi Hospital, Mahidol University, Bangkok, Thailand

**Keywords:** Ischemic stroke, Intracerebral hemorrhage, Health Outcome, Quality-adjusted life expectancy

## Abstract

**Background:**

Advanced medical technologies can prolong life of stroke survivors. Dynamic change of health outcomes provides essential information to manage stroke. Mathematical models, to extrapolate health status over a lifetime from cross-sectional data, can be used to investigate long term health outcomes among stroke survivors. This study aimed to estimate the health outcomes of ischemic stroke (IS) and intracerebral hemorrhage (ICH) at each survival time point.

**Methods:**

The cohort of 5391 patients with IS and ICH stroke, registered at Ramathibodi Hospital from 2005 to 2013, were followed up regarding survival status until 2016 with the National Mortality Registry. Survival functions were extrapolated over 50 years to age- and sex-matched referents simulated from the national data of the Thailand National Health Statistic Office. From July to December 2016, the EuroQoL 5-dimension questionnaire was used to measure quality of life (QoL) among 400 consecutive, cross-sectional subsamples. The survival functions were then adjusted by the utility values of QoL for the stroke cohort to estimate quality adjusted life expectancy (QALE).

**Results:**

The average health utility values were lower in the initial months, then slowly increased to stable levels. However, male stroke survivors presented higher health utility than females. Throughout lifetime estimation, patients with IS stroke exhibit better health outcomes than those with ICH [10.2 vs. 7.5 quality-adjusted life years (QALYs)]. Patients with ICH presented a significantly decreased QoL than patients with IS (16.3 and 8.5 QALYs).

**Conclusion:**

Preventing stroke can save people from reduced years and QoL, which can be quantified by loss-of-QALE in QALY units to compare health benefits from prevention, clinical diagnosis and direct treatment.

## Background

Stroke is the world’s second cause of death and the third cause of disability [1, 2]. During the past decades, stroke has become a global epidemic illness. From 1990 to 2013, two main types of stroke, i.e., ischemic (IS) and hemorrhagic stroke have presented an upsurge of incidence by 2 and 9%, of death by 23 and 11%, and of disability-adjusted life years (DALYs) by 31 and 15%, respectively [1].

Stroke causes not only premature death [3, 4] but compromises quality of life (QoL) among stroke survivors [5, 6]. More than one half of stroke survivors live with neurologic impairment requiring continuous and costly treatment and care to support daily living activities [6, 7]. Advanced medical technologies can prolong stroke survivors' lives, even as bedridden patients for years or decades. Such consequences likely result in catastrophic health expenditures, lost productivity within a country [8] and may grow beyond expectations in countries with aged populations lacking effective stroke prevention programs. Understanding the impacts of stroke on human health and considering stroke-related death and disability, may not reflect the real effects of stroke. QoL and lifetime utility after stroke should also be considered.

One way of examining trade-offs between survival time and QoL is to combine them in a single measure of quality-adjusted life years (QALY) [9, 10]. QALY is the health outcome measure widely used to study the burden of diseases and cost-effectiveness in healthcare services.^3, 10^ Estimating QALY requires an approximation of quality-adjusted life expectancy (QALE) and expected loss-of-QALE [11, 12]. QALE is calculated using the lifetime survival of patients with stroke adjusted by the corresponding QoL function consistent with disease occurrence duration based on the follow-up period with 50 years of extrapolation. The QALE estimation is expressed in the equation below [3, 11, 12].

$$QALE = \mathop \smallint \limits_{0}^{\infty } E\left[ {qol\left( {t|x} \right)} \right]S\left( {t|x} \right)dt$$

where $S\left( {t{|}x} \right)$ denotes the survival function for condition $x$ at time $t$, and $E\left[ {qol\left( {t|x} \right)} \right]$ denotes the expected value of health state (QoL) for patients with condition $x$ at time $t$. The loss-of-QALE is the difference between the QALE of patients and that of age- and sex-matched referent of the general population, which can be simulated from national vital statistics or life tables. Because loss-of-QALE has already been adjusted for different age distributions [13], it would be useful to directly compare potential savings from the effective prevention of two main types of illnesses, i.e., IS versus intracerebral hemorrhage (ICH).

The health utility of QoL is commonly measured using the multi-attribute utility theory, usually depending on the degree of disability or dependence on activities and instrumental activities in patient daily life, including stroke survivors. This study estimated the QALE and loss-of-QALE of both IS and cerebral hemorrhage to compare potential savings from preventing the two main types of stroke, which would also pave the way for a direct comparison of cost-effectiveness to prevent and clinically manage stroke in the future.

## Methods

A cohort of 5634 patients with stroke were abstracted from the database of Ramathibodi Hospital, a Thai medical center of tertiary care with valid diagnoses of different subtypes of stroke. The estimations included three steps. First, we developed the lifetime survival function by linking the survival function of patients with stroke and Thai national life tables. Second, we generated the QoL function by measuring QoL among the consecutive, cross-sectional subsample of the stroke cohort and, finally, estimated the QALE and loss-of-QALE by combining the two functions.

### Extrapolation of the survival function to lifetime

Firstly, we generated the 9-year follow-up stroke cohort to estimate the survival function. The data of the first-ever stroke from the database of Ramathibodi Hospital, Bangkok, Thailand, from January 2005 to December 2013 were included. Of 5634 stroke cases, 105 cases (1.86%) with incomplete data on survival time, and 138 stroke cases (2.45%) experiencing other significant comorbidities were excluded from this study to control potential confounding of mortality from other causes. Of 138 excluded stroke cases, there were 47 cases of lung cancer, 31 cases of liver cancer, 6 cases of pancreatic cancer, 36 cases of leukemia, and 18 deaths of heart failure. The survival status of patients with stroke was verified by cross-validating with the National Mortality Registry. Finally, 5391 stroke cases remained in this cohort were followed up their survival status until the end of 2016, which included both who died rapidly after having a stroke and long-term survivors. In fact, the numbers of stroke patients in the cohort who died within day 1, 7, and 30 days were 66, 370, and 688 cases, respectively. Then we estimated the survival function for two main stroke subtypes (ischemic stroke and intracerebral hemorrhage) using the Kaplan–Meier method.

We applied a semi-parametric extrapolation method to estimate the extra 50 years from survival function based on Kaplan–Meier method estimations. The lifetime survival function was estimated by incorporating the life expectancy (LE) information from an age- and sex-matched referent population in the estimation process of 144 months. The estimates were acquired using an open access iSQoL Software [14]. This extrapolation method was confirmed as a reliable method to generate the lifetime survival after the follow-up limit [3, 15–17].

### QoL measurement of a consecutive, cross-sectional subsample

To estimate the average QoL along different time t, we measured QoL among consecutive cross-sectional subsamples of the stroke cohort [11]. Regarding the 5-dimension EuroQol questionnaire (EQ-5D-5L) [18], the Thai version was used to measure QoL in terms of utility values in each state of time of individuals with stroke. The utility values ranged from 0 to 1 (0, death, and 1, perfect health). The time length since the first-stroke diagnosis until the QoL assessment date was assumed to be time t.

Patients with stroke from the inpatient stroke unit and the outpatient neurologic clinics, Ramathibodi Hospital, from July 2016 to December 2016, were invited to participate in this study. We excluded 15 cases, 11 cases did not cognitively communicate and 4 cases quit while being interviewed. We prospectively measured QoL among the 387 consecutive cross-sectional subsamples. Some individuals (13 cases) had QoL measured repeatedly, however, we included only the first measurements in our analysis.

In the estimating process for the QoL function, we applied a Kernel smoothing method to estimate the average health-related QoL (HRQoL) function, which uses the moving average of the neighboring 10% [11, 12]. The QoL utility values beyond the follow-up period were assumed to be the same as the average of the last 10% of patients near the end of follow-up. For each time t, the mean utility value of stroke survivors was multiplied with the survival rate of the cohort, whereas the utility for all hypothetical referents was assumed to be 1 throughout the survival period.

### Estimation of QALE and loss-of-QALE

The QALE constituted the expected lifetime full utility after developing a stroke. The lifetime survival function of patients with stroke adjusted by the corresponding mean QoL function, consistent with the duration of stroke occurrence, was used to calculate QALE based on 12-year follow-up period with 50 years of extrapolation matched-paired by age and sex. We estimated the loss of QALE by subtracting the area under the survival curve of patients with the stroke from the age- and sex-matched referents.

We further performed a stratified analysis among patients with a degree of disability using the same methods. The degree of disability was classified using modified Rankin Score (mRS), scoring 0–2 as independent and 3–5 as disabled [19]. The lifetime utilities of disabled and independent stage patients were estimated.

## Results

### Sample description

Table 1 summarizes the characteristics of patients with stroke from the cohort and cross-sectional subsample to analyze survival function and measure the QoL. A total of 5391 individuals with ischemic stroke (IS) and intracerebral hemorrhage (ICH) in the Ramathibodi stroke registry from 2005 to 2013 were enrolled in the cohort study. About 54.4% were male. Of 5,391, 73.3% were patients with IS, and the rest were patients with ICH. The average age of all study subjects was 62.5 years old (SD = 17.9). Mean age among individuals with ICH (56.1 years; SD = 20.7) was younger than that of individuals with IS (64.7 years; SD = 16.1).

**Table 1:** Individual characteristics of patients with stroke between stroke cohort data and subsample from Ramathibodi Hospital

	Stroke cohort (n = 5391)	Subsample (n = 387)	*p* Value
Onset of stroke	2005–2013	2000–2016	
Age, years, means (SD)	62.5 (17.9)	66.6 (13.4)	< 0.001
Male, %	54.4	49.9	0.086
Ischemic stroke, number (%)	3,950 (73.3)	314 (81.1)	0.001
Intracerebral hemorrhage, number (%)	1,441 (26.7)	73 (18.9)	
*Ischemic stroke*			
Age, years, means (SD)	64.7 (16.1)	66.4 (13.6)	0.039
Male, < 65 years, %	25.5	21.7	0.412
Male, ≥ 65 years, %	27.2	27.1	
Female, < 65 years, %	17.8	20.1	
Female, ≥ 65 years, %	29.5	31.1	
*Intracerebral hemorrhage*			
Age, years, means (SD)	56.1 (20.7)	67.3 (12.4)	< 0.001
Male, < 65 years, %	40.7	26.0	0.002
Male, ≥ 65 years, %	18.1	28.8	
Female, < 65 years, %	20.4	12.3	
Female, ≥ 65 years, %	20.7	32.9	
*Comorbidities*^*x*^*, %*			
Diabetes mellitus, %	28.0	32.6	0.053
Hypertension, %	58.1	74.1	< 0.001
Dyslipidemia, %	33.0	53.6	< 0.001
Atrial fibrillation, %	15.1	19.2	0.030
Coronary artery disease, %	10.4	15.3	0.003
Lung disease, %	1.8	1.6	0.707
Renal disease, %	14.8	9.8	0.008
Charlson comorbidity index, median (IQR)	2 (3)	2 (2)	0.060

^x^Multiple choices

*p* Values: Independent t-test for quantitative data and Chi-square test for qualitative data

*SD* standard deviations, *IQR* interquartile range

A consecutive cross-sectional subsample included 387 patients with IS and ICH. The mean age was 66.4 years for patients with IS and 67.3 years for ICH. The ratio of male and female respondents was 1:1.

The individual characteristics of patients with stroke between cohort and cross-sectional subsample significantly differed by age, and type of stroke. The proportion of comorbidities of the two sets of data differed. However, the Charlson comorbidity index (CCI), referring to the risk of mortality, did not differ between these two data sets. To control confounder cross-sectional subsample, the QALE was stratified analyzed by types of stroke and matched by age and sex.

### Main findings

**Fig. 1:**
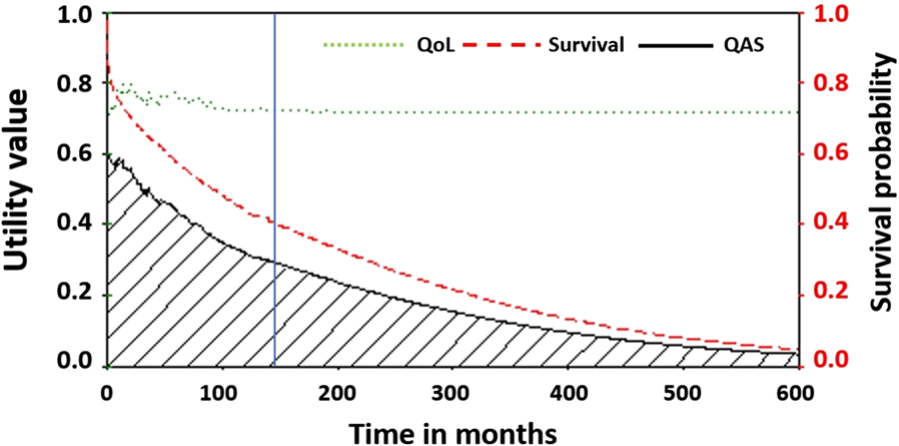
The survival probability, average QoL score, and quality-adjusted survival of patients with stroke

The characteristics of QoL-interviewed respondents are summarized in Table 2. The mean duration since the first stroke was diagnosed until the QoL assessment date was 2.26 years. The average utility value of QoL in IS (0.74) was higher than that in ICH (0.69). Also, the better conditions were also found among males (0.78) than among females (0.68), and the patterns were similar between IS and ICH (Table 2). The degree of disability of daily living was directly related to the level of QoL concerning utility values. Figure 1 demonstrates the estimated survival, average QoL score, and the quality-adjusted survival (QAS) function for patients with stroke over 144 months, then extrapolating to 600 months. The average utility values of QoL were lower in the initial months, then slowly increased to a stable level.

**Table 2:** Results of quality of life (QoL) assessments and estimated loss of quality-adjusted life expectancy (QALE)

Characteristics	Overall	IS	ICH
	Mean (SD)	Mean (SD)	Mean (SD)
EQ-5D-interviewed cases, number (%)	387 (100)	314 (81.1)	73 (18.9)
Time elapsed since the first stroke, years	2.26 (2.66)	2.16 (2.53)	2.71 (3.13)
Utility value of QoL	0.73 (0.26)	0.74 (0.25)	0.69 (0.32)
Male	0.78 (0.24)	0.79 (0.21)	0.72 (0.31)
Female	0.68 (0.28)	0.69 (0.26)	0.65 (0.33)
*Utility values classified by BI*			
Independent (BI 100)	0.87 (0.13)	0.86 (0.13)	0.93 (0.09)
Slight dependent (BI 75–95)	0.67 (0.11)	0.66 (0.09)	0.72 (0.13)
Moderate dependent (BI 50–75)	0.51 (0.12)	0.50 (0.14)	0.54 (0.04)
Severe dependent (BI 25–45)	0.24 (0.12)	0.26 (0.13)	0.20 (0.06)
Total dependent (BI 0–20)	0.13 (0.05)	0.12 (0.05)	0.14 (0.05)
*Utility values classified by mRS*			
0	0.90 (0.12)	0.89 (0.12)	0.95 (0.09)
1	0.85 (0.15)	0.85 (0.15)	0.90 (0.14)
2	0.82 (0.18)	0.81 (0.18)	0.88 (0.13)
*0–2 (independent)*	*0.85 (0.16)*	*0.84 (0.16)*	*0.90 (0.13)*
3	0.68 (0.25)	0.70 (0.24)	0.62 (0.30)
4	0.62 (026)	0.64 (0.24)	0.53 (0.34)
5	0.39 (0.29)	0.38 (0.26)	0.40 (0.33)
*3–5 (disabled)*	*0.59 (0.29)*	*0.61 (0.27)*	*0.52 (0.33)*
Estimation of LE and QALE	Mean (SE)	Mean (SE)	Mean (SE)
LE, years	13.59 (0.85)	13.25 (1.02)	13.21 (1.77)
EYLL, years	6.62 (0.47)	5.51 (0.66)	10.66 (1.37)
QALE, QALYs	9.94 (0.39)	10.35 (0.31)	7.72 (1.02)
Loss-of-QALE relative to referents, QALYs	10.27 (0.36)	8.41 (0.28)	16.15 (0.98)

The unit of QALE is quality-adjusted life year (QALY)

A *p *Value < 0.05 is considered statistically significant

*BI* Barthel index, mRS modified rankin score, *LE* life expectancy, *EYLL* expected years of life lost, *QALE* quality-adjusted life expectancy, *SD* standard deviation, *SE* standard error

**Fig. 2:**
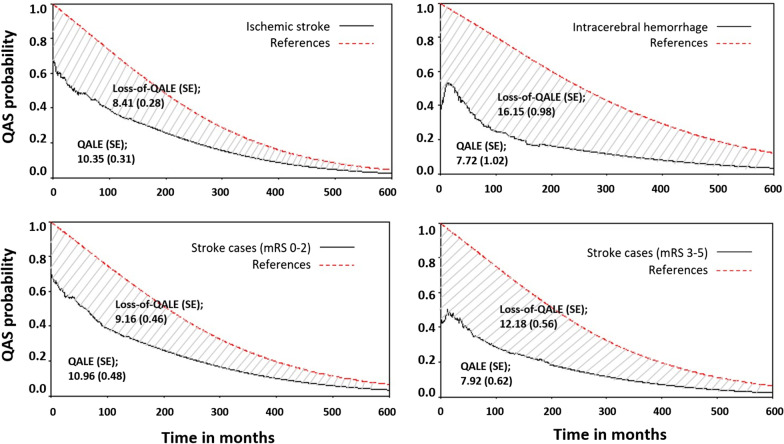
Quality-adjusted survival curves of patients and the age- and sex-matched referents stratified by stroke types and disabled state

The QAS probability throughout the patient’s lifetime was estimated by multiplying lifetime survival function and QoL function (Fig. 2). The lifetime survival function started from 1 at the beginning time (t = 0) and smoothly declined toward 0 as time increased toward infinity. The shaded area under the QAS curve represents the expected cumulative QALE throughout lifetime after stroke.

QoL stands for quality of life (the green dotted line). Survival stands for survival proportion of stroke cohort (red dashed line). QAS stands for quality adjusted survival (blackish line). The follow-up time ended at 145 months (indicated by blue line), then extrapolating to 6oo months. The shaded area represents the expected cumulative QALE (quality-adjusted life expectancy) throughout life after stroke which is the area under the QAS curve.

Y-Axis is the quality-adjusted survival (QAS) probability. The shaded area is the loss-of-QALE (quality-adjusted life expectancy). QALE of the referent population without stroke assumed utility to be 1. QALY stands for quality-adjusted life year; mRS stands for modified Rankin Scores. (Number in parenthesis is the standard error of mean).

**Fig. 3:**
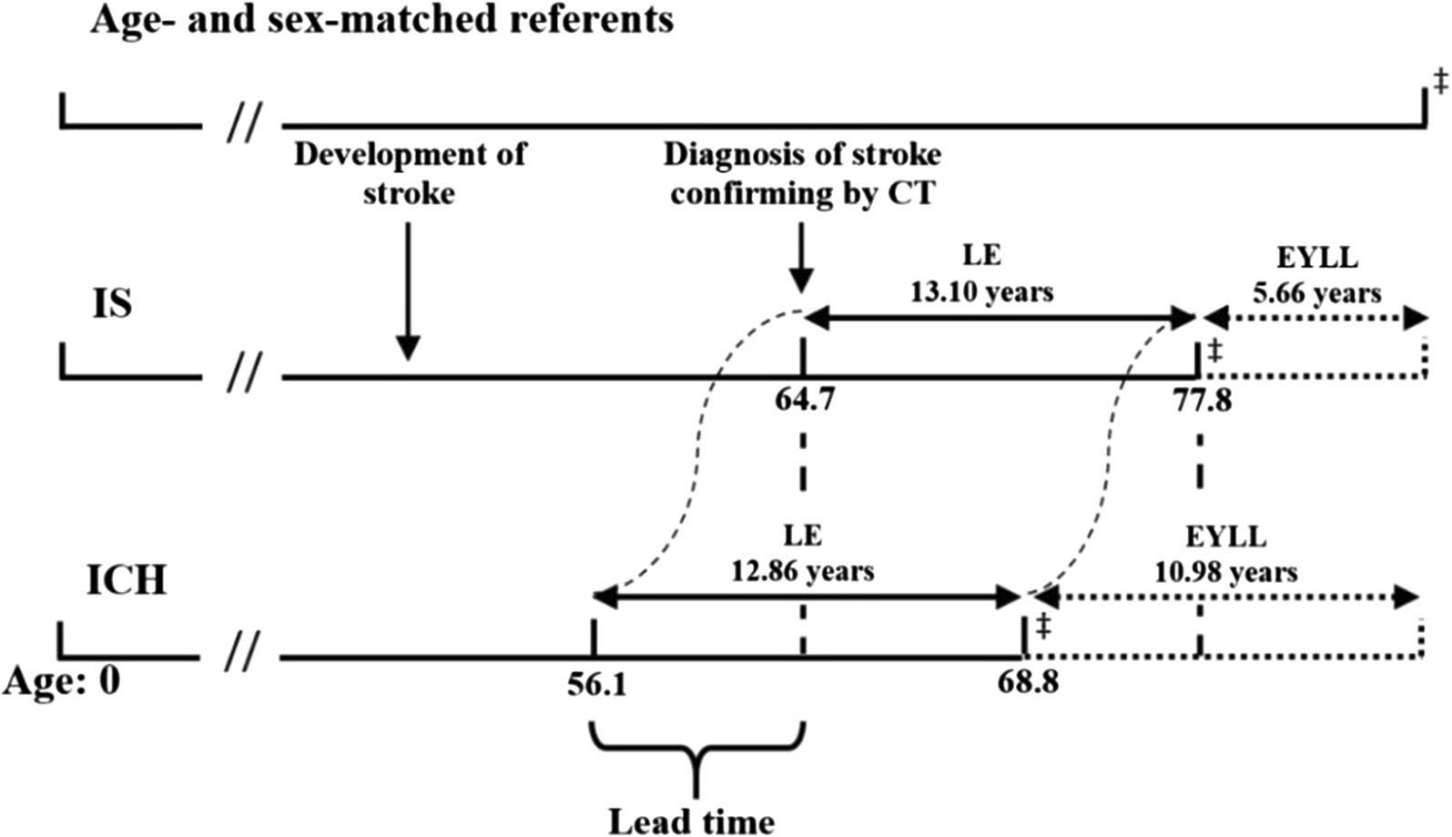
Adjustment for different age and sex distributions between the two sub-cohorts

The EYLL and loss-of-QALE, as stratified by types of stroke, are summarized in Table 2. The average life expectancy of patients with stroke was 13.59 years. The average life expectancy of a patient with IS and ICH were similar (13.25 years and 13.21 years, respectively). However, patients with ICH occurred at a younger age than of patients with IS, leading to a longer life expectancy for ICH. However, ICH resulted in a more extensive life loss and loss-of-QALE. The EYLL was adjusted for different ages at diagnosis; thus, the difference of EYLLs would also be adjusted for that confounder. Figure 3 displays how lead-time bias would be adjusted by comparing the differences in EYLLs for different ages at diagnosed IS versus ICH. After adjusting for lead-time bias between two ages of diagnoses, the potential gain of prevention for ICH and IS would be 10.66 and 5.51 years, respectively (Table 2).

Average age of patients with a diagnosis of intracerebral hemorrhage (ICH) is on average 8.6 years younger than those with ischemic stroke (IS). CT stands for computed tomography. LE stands for life expectancy. EYLL stands for expected years of life lost. ^**‡**^ indicates death.

The average QALE of patients with stroke was estimated to be 9.94 QALYs. Patients with IS were expected to have a better QALE than patients with ICH (10.35 vs. 7.72). The loss-of-QALE relative to the age- and sex-matched referent was 10.27 QALYs. Patients with ICH had a significantly higher loss of 7.74 QALYs than those with IS (16.15 QALYs vs. 8.41 QALYs). Because of the onset at a younger age, the QAS function of the referents matched for patients with ICH was higher than that of the referents matched for patients with IS. It resulted in a more significant gap between the stroke and the referent populations (Fig. 2).

We also performed a stratified analysis among patients with stroke and a degree of disability (Fig. 2). Compared with the disabled stage, patients at the independent stage had a significantly longer QALE (10.96 versus 7.92 QALYs). Moreover, the loss-of-QALE for patients at the disabled stage was 3.04 years greater than that of patients at the independent stage.

## Discussion

To assure the quality of diagnosis for different subtypes of stroke, we collected all the patients of the stroke cohort from a medical center of tertiary care. Unlike related studies using internationally chosen life tables and the expert’s agreement of disability weights to calculate the disease burden of stroke for Thailand using DALY [20, 21], we analyzed these real world data to provide evidence of a more detailed estimation of burden of stroke, or, with a unit of QALY (quality-adjusted life year) [3, 10, 17]. Moreover, we have stratified subtypes of stroke and integrated them with lifetime survival function to obtain QALE. Some studies measured cross-sectional HRQoL for patients with stroke [6, 7, 22], but lifetime survival function was not considered. Our study seems the first to consider both survival and QoL to estimate the QALE of the stroke cohort of a medical center of tertiary care in Thailand.

Moreover, we also estimated loss-of-QALE to adjust for different age distributions of different sub-cohorts with hemorrhage versus infarction, as illustrated in Fig. 3. In other words, the two different ages of diagnosis between IS and ICH were considered by counting the loss-of-LE instead of LE, and we have demonstrated that the loss-of-QALE is 7.74 (16.15 minus 8.41) QALY more in hemorrhage instead of the apparent 2.69 (10.35 minus 7.72) QALY. We thus concluded that the loss of lifetime utility is much greater in ICH than in IS and deserves more efforts in prevention.

This study found that, on average, a patient would lose 10.27 QALYs due to stroke in Thailand, which is greater than patietns with stroke in Taiwan and Australia [3, 23]. The expected lifetime loss of utility from ICH was found to be almost 8 QALYs more than that from IS, which can be attributed to the younger age of onset of ICH (8.60 years). This difference may result from the finding that ICH had worse health outcomes than IS during the early periods of follow-up [24]. The lower rate of survival at the early period of patients with ICH affected the estimation of life expectancy and QALE, resulting in poorer ICH outcomes [25]. The nature of disease and treatment differs between IS and ICH [24]. ICH occurs when a reduced or lack of blood flow to the brain results in impaired brain functions. However, appropriate treatment during the acute period using thrombolytic agents, antiplatelets, or anticoagulants to open the vessels and normalize perfusion could save the penumbra and limit brain damage [26]. Intracerebral hemorrhage, on the other hand, is caused by the rupture of a blood vessel. The rupture not only cuts off the connecting pathway to the brain, but bleeding also directly goes through the brain tissue, compressing the nearby brain area, increasing intracranial volume, and elevating intracranial pressure. These mechanisms can lead to severe outcomes [24]. Treatment options for ICH involve providing supportive treatment and controlling of the expansion of bleeding [27]. Removing the blood clot and reducing increased intracranial pressure can be managed by early brain surgery; however, a comprehensive surgical approach is not a promise of good results and can lead to further brain damage or increased bleeding [27].

The lifetime utilities among patients with disabled and independent states were identified in this study. Patients who are disabled were expected to lose 3.04 years of perfect health utility compared with those with an independent state. The greater the severity the patient faces, the higher the burden they experience [19]. Appropriate early interventions are suggested to limit damage from stroke. Moreover, appropriate rehabilitation programs and long-term care should be provided to patients to improve their health functions and reduce the burden of stroke.

This study had the advantages of a large cohort with homogenous diagnostic criteria and 12 years of follow-up. All stroke cases in this study were confirmed by brain imaging (CT scan and/or MRI); thus, we could be certain that the stroke diagnoses and classifications were accurate. Survival status of all cases was verified through cross-validation with the National Mortality Registry, providing the exact death date of the study population resulting in identifying precise time-periods of survival after stroke. However, this study identified death based on all causes of death. It could have led to underestimating survival for patients with stroke who died from other significant diseases or accidents.

To the best of our understanding we also obtained the EQ-5D-5L assessed from bedridden patients to the best of our understanding regarding the burden of stroke. Therefore, the QoL functions covered a wide range of stroke consequences, except for those with major cognitive impairment. Thus, the estimations of QoL functions might have been underestimated the overall burden of stroke. The study aimed to capture the trend of QoL from the acute to the chronic phase of the stroke. The EQ-5D-5L assessments were conducted over a six-month timeframe resulting in some limitations. First, the mean QoL value reported by this study sample (0.73) appeared to be on the edge of QoL ranges from some other studies (0.50–0.81) [3, 28]. In addition, the six-month period of our data collection might have provided us with more chances to recruit patients with more improved status than those in the three-month timeframe data collection [28].

Limitations were encountered in this study. Because we were unable to performed measurement on patients with major cognitive impairment, we did not include such patients and our study likely underestimated the loss-of QALE among stroke survivors. Future studies are warranted to further stratify them into more detailed subtypes, such as arterial atherosclerosis, cardio embolism, and lacuna stroke, etc. in the stroke cohort and evaluate effects of different technology of healthcare services.

## Conclusions

This study successfully estimated the QALE and loss-of-QALE of patients with stroke and ICH as well as the loss between disabled and independent states. The potential gain of prevention would be up to 16.15 QALYs if we could prevent people from developing ICH and 8.41 QALYs from IS. In conclusion, stroke prevention programs are suggested to help people avoid developing stroke in the first place. The loss-of-QALE is an appropriate health outcome measure providing evidence-based guidance needed to improve care and prevention programs.

## Data Availability

The datasets generated and analyzed during the current study are not publicly available because they constitute an excerpt of research in progress but are available from the corresponding author on reasonable request.

## Abbreviations

BI: Barthel index
CCI: Charlson comorbidity index
DALY: Disability-adjusted life years
EYLL: Expected years of life lost
EQ-5D-5L: EuroQol questionnaire
HRQoL: Health-related QoL
ICH: Intracerebral hemorrhage
IS: Ischemic stroke
IQR: Interquartile range
mRS: Modified Rankin Score
QALE: Quality-adjusted life expectancy
QALY: Quality-adjusted life years
QoL: Quality of life
SD: Standard deviation
SE: Standard error
