# Correction to: Combined quality of life and survival for estimation of long-term health outcome of patients with stroke

**DOI:** 10.1186/s12955-022-01983-1

**Published:** 2022-05-03

**Authors:** Nipaporn Butsing, Mathuros Tipayamongkholgul, Jung-Der Wang, Disya Ratanakorn

**Affiliations:** 1 Ramathibodi School of Nursing, Faculty of Medicine Ramathibodi Hospital, Mahidol University, Bangkok, Thailand; 2 Department of Epidemiology, Faculty of Public Health, Mahidol University, 420/1 Ratchawithi Road, Ratchathewi, Bangkok, 10400 Thailand; 3 Department of Public Health, National Cheng Kung University, College of Medicine, Tainan City, Taiwan; 4 Department of Medicine, Faculty of Medicine, Ramathibodi Hospital, Mahidol University, Bangkok, Thailand

## Correction to: Health and Quality of Life Outcomes (2022) 20:46 https://doi.org/10.1186/s12955-022-01959-1

The original article [1] contained errors in the Abstract which have since been corrected.
